# ﻿A new species of terrestrial toad of the *Rhinellafestae* group (Anura, Bufonidae) from the highlands of the Central Cordillera of the Andes of Colombia

**DOI:** 10.3897/zookeys.1196.114861

**Published:** 2024-03-25

**Authors:** Luis Santiago Caicedo-Martínez, Jose J. Henao-Osorio, Héctor Fabio Arias-Monsalve, Julián Andrés Rojas-Morales, Paula A. Ossa-López, Fredy A. Rivera-Páez, Héctor E. Ramírez-Chaves

**Affiliations:** 1 Natural History Laboratory, Integrative Zoological Biodiversity Discovery, Centro de Museos, Museo de Historia Natural, Universidad de Caldas, Carrera 23 # 58-65, Manizales 170004, Colombia; 2 Programa de Biología, Facultad de Ciencias Exactas y Naturales, Universidad de Caldas, Calle 65 No. 26-10, Manizales 170004, Colombia; 3 Fundación Ecológica Cafetera, Manizales, Caldas, Colombia; 4 Grupo de Investigación en Genética, Biodiversidad y Manejo de Ecosistemas (GEBIOME), Departamento de Ciencias Biológicas, Facultad de Ciencias Exactas y Naturales, Universidad de Caldas, Calle 65 No. 26-10, Manizales 170004, Colombia

**Keywords:** Andes, Central Cordillera, distribution, diversity, endemism, systematics

## Abstract

The genus *Rhinella* (Bufonidae) comprises 92 species of Neotropical toads. In Colombia, *Rhinella* is represented by 22 recognized species, of which nine belong to the *Rhinellafestae* group. Over the past decade, there has been increasing evidence of cryptic diversity within this group, particularly in the context of Andean forms. Specimens of *Rhinella* collected in high Andean forests on both slopes of the Central Cordillera in Colombia belong to an undescribed species, *Rhinellakumanday***sp. nov.** Genetic analyses using the mitochondrial 16S rRNA gene indicated that the individuals belong to the *festae* species group. However, they can be distinguished from other closely related species such as *Rhinellaparaguas* and *Rhinellatenrec* by a combination of morphological traits including the presence of tarsal fold, a moderate body size, and substantial genetic divergence in the 16S rRNA gene (> 5%). Through this integrative approach, the specimens from the Central Cordillera of Colombia are considered an evolutionary divergent lineage that is sister to *R.paraguas*, and described as a new species. *Rhinellakumanday***sp. nov.** is restricted to the Central Cordillera of Colombia inhabiting both slopes in the departments of Caldas and Tolima, in an elevational range between 2420 and 3758 m. With the recognition of this new species, the genus *Rhinella* now comprises 93 species with 23 of them found in Colombia, and ten species endemic to the country.

## ﻿Introduction

*Rhinella* Fitzinger, 1826 (Bufonidae) comprises 92 species of toads distributed mainly in the Neotropical region (Pereyra et al. 2021; Frost 2023). Over the past decades, several studies have delved into the taxonomic and phylogenetic complexity of the genus, resulting in the description of new species (Pramuk 2006; Moravec et al. 2014; Castillo-Urbina et al. 2021; Lehr et al. 2021), and the systematics examination of specific species groups (Graybeal 1997; Maciel et al. 2006; Pramuk 2006; Chaparro et al. 2007; Fouquet et al. 2007; Narvaes and Rodrigues 2009; Pyron and Wiens 2011; Moravec et al. 2014; Pereyra et al. 2016; 2021; Rivera et al. 2021). In a more recent and comprehensive study of the systematics of *Rhinella*, Pereyra et al. (2021) defined eight distinct species groups based on a combination of osteological, muscular, external morphology, and molecular data: *R.arunco*, *R.crucifer*, *R.festae*, *R.granulosa*, *R.margaritifera*, *R.marina*, *R.spinulosa*, and *R.veraguensis*.

The *festae* group was first proposed based on genetic data by Moravec et al. (2014) and initially consisted of seven species. This group incorporated three species from the *acrolopha* group (including taxa previously considered within the genus *Ramphophryne* Trueb, 1971): *R.festae* (Peracca, 1904), *R.macrorhina* (Trueb, 1971), and *R.rostrata* (Noble, 1920). Additionally, the *festae* group included four species from the paraphyletic *R.veraguensis* group: *R.manu* Chaparro, Pramuk & Gluesenkamp, 2007, *R.nesiotes* Duellman & Toft, 1979, *R.chavin* Lehr, Köhler, Aguilar & Ponce, 2001, and *R.yanachaga* Lehr, Pramuk, Hedges & Córdova, 2007. More recently, Pereyra et al. (2021) redefined the *festae* group, which now comprises 20 species, including two yet-to-be described species from Colombia and Peru. The undescribed species from Colombia was identified based on a single specimen collected at the Central Cordillera in the Andean region of the Department of Tolima (Pereyra et al. 2021).

Pereyra et al. (2021) expanded the *festae* group by reclassifying ten species that were previously part of the *acrolopha* group from Colombia, Ecuador, Panama, and Peru. Furthermore, Pereyra et al. (2021) resolved the paraphyly of the *veraguensis* group by reassigning eight species from Bolivia and Peru in the *festae* group. As a result of these changes, together with the addition of recently described species have brought the *festae* group to a total of 22 species (two remaining undescribed) including: i) *R.acrolopha* (Trueb, 1971), ii) *R.arborescandens* (Duellman & Schulte, 1992), iii) *R.chavin*, iv) *R.chullachaki* Castillo-Urbina, Glaw, Aguilar-Puntriano, Vences & Köhler, 2021, v) *R.lilyrodriguezae* Cusi, Moravec, Lehr & Gvozdik, 2017, vi) *R.festae*, vii) *R.lindae* (Rivero & Castaño, 1990), viii) *R.macrorhina*, ix) *R.manu*, x) *R.moralesi* Lehr, Cusi, Rodríguez, Venegas, García-Ayachi & Catenazzi, 2021, xi) *R.multiverrucosa* (Lehr, Pramuk & Lundberg, 2005), xii) *R.nesiotes*, xiii) *R.nicefori* (Cochran & Goin, 1970), xiv) *R.paraguas* Grant & Bolívar-G., 2014, xv) *R.rostrata*, xvi) *R.ruizi* (Grant, 2000), xvii) *R.tacana* (Padial, Reichle, McDiarmid & De La Riva, 2006), xviii) *R.tenrec* (Lynch & Renjifo, 1990), xix) *R.truebae* (Lynch & Renjifo, 1990), xxi) *R.yanachaga*, and the two undescribed species, one each from Peru and Colombia in Pereyra et al. (2021).

In Colombia, *Rhinella* is represented by 22 recognized species, nine of which belong to the *festae* group (Pereyra et al. 2021). Most of these species are primarily found in restricted montane regions, in elevations ranging from moderate to highland altitudes (1400–3100 m a.s.l) in the Andes. The exception to this pattern is *R.acrolopha* which is distributed in the Biogeographic Chocó Region (Trueb 1971; Lynch and Renjifo 1990; Grant and Bolívar-G 2014; Pereyra et al. 2021). Among the *festae* group, one of the two undescribed species occurs in the Central Cordillera of Colombia, along the road between the cities of Manizales in the Department of Caldas (Rojas-Morales et al. 2014), and Murillo, Department of Tolima (Machado et al. 2016; Pereyra et al. 2021). In the last two decades, individuals tentatively assigned to this undescribed species have been recorded in the highlands of the municipality of Villamaría, Caldas, in close proximity to the previously reported localities (see Rojas-Morales et al. 2014; Rojas-Morales and Marín-Martínez 2019). After conducting a meticulous revision of the specimens from the Central Cordillera of the Department of Caldas using morphological and genetic analyses, we found that they possess distinct morphological and genetic characteristics that warrant their classification as a new species. This new species is exclusively found in Andean ecosystems that are highly threatened. Recognizing the taxonomic status of these populations is crucial for effective conservation planning. Therefore, in this study, we formally describe the specimens from the Central Andes in the departments of Caldas and Tolima, previously categorized within the *festae* group, based on genetic analyses and discrete external and osteological traits.

## ﻿Materials and methods

To morphologically diagnose the new species, we reviewed specimens of *Rhinella* from the Central Cordillera of Colombia, deposited in the Colección de Anfibios of the Museo de Historia Natural of the Universidad de Caldas (**MHN-UCa-Am**), and a collection of new specimens, which were euthanized with 5% lidocaine, fixed in 10% formalin and stored in 70% ethanol. The diagnosis follows the proposal by Trueb (1971) and Grant and Bolívar-G. (2014); the osteological description was carried out based on two double stained and cleared specimens (MHN-UCa-Am 1492, 1802). For this procedure, we followed the protocol proposed by Taylor and Van-Dyke (1985) and the modifications made by Fernández-Blanco and Witmer (2020); osteological terminology and characters were taken from Pramuk (2006) corrected by Pereyra et al. (2021), as well as those reported by Deforel et al. (2021). We also used the species description of Vélez-R and Ruiz-C (2002), Grant and Bolívar-G. (2014) and Castillo-Urbina et al. (2021) for comparison. We described the webbing formulae following to Savage and Heyer (1967) and the modifications by Myers and Duellman (1982) and Savage and Heyer (1997). Fingers were counted from pre-axial to post-axial side as I–IV in hand and I–V in foot; the comparative length of fingers I and II was taken by adpressing to each other, and adpressing toes III and V to toe IV (Castillo-Urbina et al. 2021). We took external measurements using a digital caliper (± 0.01 mm) following Castillo-Urbina et al. (2021) and included: Snout-vent length (**SVL**) ; Head width (**HW**) ; Head length (**HL**) ; horizontal eye diameter (**ED**) ; interorbital distance (**IOD**) ; upper eyelid width (**EW**) ; upper eyelid length (**EL**) ; internarial distance (**IND**) ; Eye-nostril distance (**E-N**) ; Nostril-snout distance (**NSD**) ; snout length (**SL**) ; forearm length (**FL**) ; hand length (**HNDL**) ; femur length (**FEML**) ; tibia length (**TL**) ; foot length (**FOOTL**) and, parotoid length (**PL**).

To compare genetic affinities of the *Rhinella* from the Central Cordillera in the Department of Caldas, we extracted genomic DNA from two specimens from tissues preserved in 96% ethanol. DNA was extracted with a Wizard® Genomic DNA Purification kit (Promega Corporation) following the manufacturer´s protocol, with modifications in the incubation time (24 h). We performed amplification of the mitochondrial 16S gene using primers 16S Ar-L 5’–CGCCTGTTTATCAAAAACAT–3’ 16S Br-H 5’-CCGGTCTGAACTCAGATCACGT-3’ which amplify a fragment of ≈520 bp (Palumbi et al. 1991). The PCR products were sent to Macrogen Inc. (South Korea) for purification and sequencing. The sequences were analyzed in Geneious Prime® v. 2023.1.2 software.

A phylogenetic analysis was performed following the updated taxonomy by Pereyra et al. (2021), including the species of the *R.festae* group, except for species *R.moralesi* and *R.truebae* that lack sequences in GenBank. In total, 185 DNA sequences previously reported in GenBank were used for the analysis, with *Anaxyrusamericanus* (Holbrook, 1836), *A.woodhousii* (Girard, 1854), and *A.quercicus* (Holbrook, 1840) used as the outgroup (Suppl. material 1). Sequence alignment was performed in MAFFT v. 7 (Katoh and Standley 2013), ambiguously aligned fragments were removed using Gblocks (Talavera and Castresana 2007) included in PhyloSuite (Zhang et al. 2020). The best fit evolutionary model was selected across ModelFinder (Kalyaanamoorthy et al. 2017) using AIC criterion: TIM2+F+I+G4. Phylogenetic inferences were constructed using Maximum Likelihood (ML) in IQ-TREE v 2.2.0 (Nguyen et al. 2015) with 1000 ultrafast-bootstrap (UFB) (Minh et al. 2013), as well as the Shimodaira–Hasegawa–like approximate likelihood-ratio test (Guindon et al. 2010). We used FigTree v. 1.4.3 to visualize the phylogenetic tree (Rambaut 2007). Genetic distances were estimated using the *p*-*distance* method with the MEGA v. 11 program.

## ﻿Results

We reviewed nine specimens of the *R.festae* group deposited in the collections of the MHN-UCa-Am (including one specimen preserved as skeleton). The specimens exhibited morphological characteristics commonly associated with the *R.festae* group including the absence of the annulus tympanicus and tympanic membrane, a slightly exostosed skull; webbed fingers; a poorly developed preorbital crest, and a weak supratympanic crest. In addition to these typical traits, we also identified a set of unique morphological traits that set them apart from other *Rhinella* species distributed in the Central Cordillera and neighboring areas (i.e., *R.acrolopha*, *R.festae*, *R.lindae*, *R.macrorhina*, *R.nicefori*, *R.paraguas*, *R.rostrata*, *R.truebae*, and *R.tenrec*). The distinct traits included the presence of a tarsal fold, the extent of skull ornamentation, and the number of vertebrae (seven).

**Figure 1.**
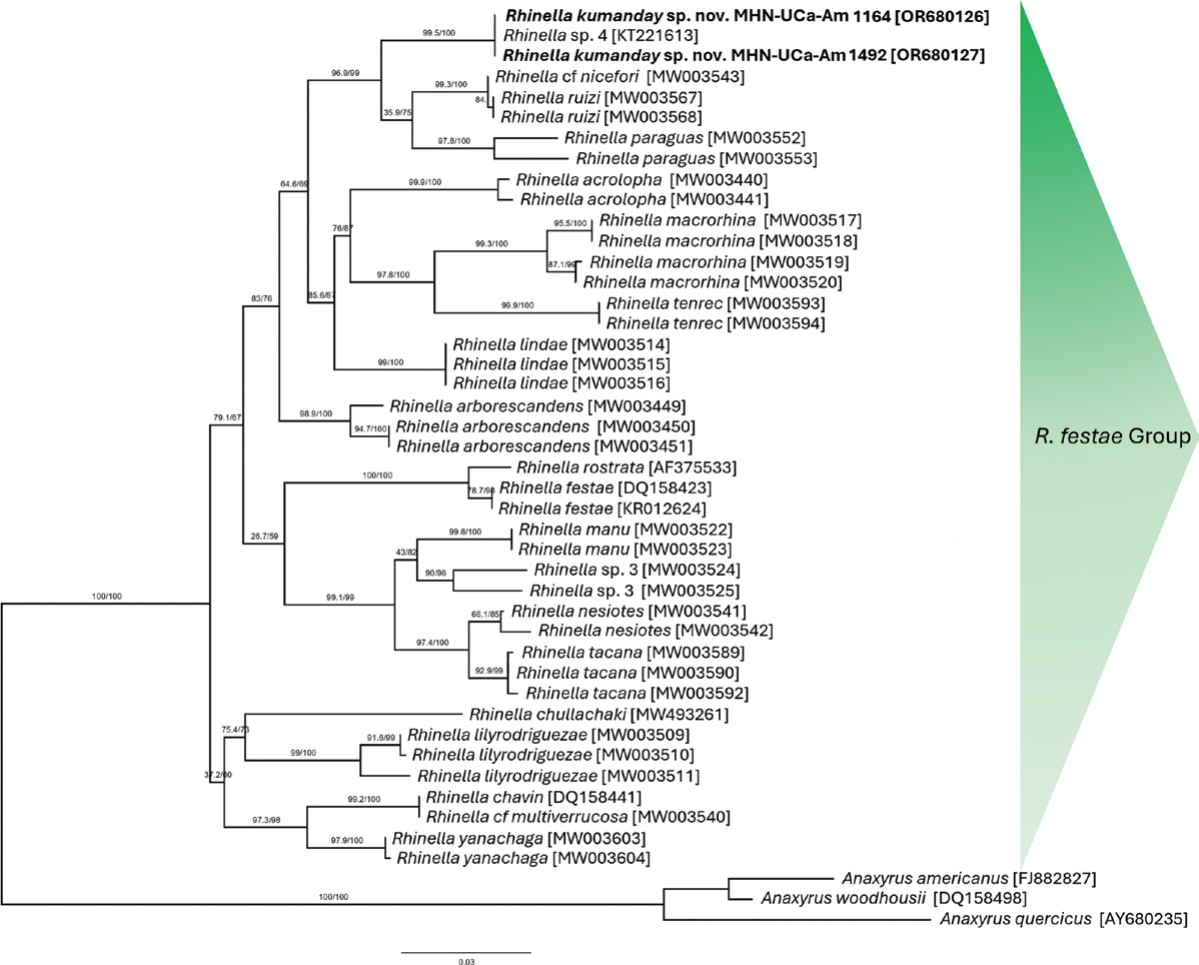
Phylogenetic tree of the partial sequences of the 16S gene of the species of *Rhinella*. The accessions in the present study (in bold), and of the sequences in GenBank (accession numbers in bracket), using maximum likelihood (ML) method under the TIM2+F+I+G4 model. Numbers at nodes are selbranch support analyses; from left to right: ultrafast bootstrap values, and Shimodaira–Haseglike approximate likelihood ratio test (SH-like aLRT). Three species of *Anaxyrus* sequences were used as outgroups.

For the genetic comparisons, we generated two new mitochondrial *16S* rDNA sequences [accession numbers: OR680126; OR680127] from two specimens (MHN-UCa-Am 1164 and 1462). Both sequences exhibit a 100% similarity with a single sequence [KT221613] identified as *Rhinella*. sp. “gr. acrolopha” (Pereira et al. 2021: 112) or “*Rhinella* sp. 4” originating from a specimen collected at in Murillo, km 22 road Murillo-Manizales, Department of Tolima. The collection or museum in which the specimen from which the sequence KT221613 was obtained is deposited is unknown. The interspecific genetic distances (Table 1) between the specimens of *Rhinella* from the Central Cordillera and other species within the *R.festae* group (except for *R.moralesi* and *R.truebae*) ranged from 3.6% to 10.1% for the *16S* gene (Table 1) and are within the ranges known for different species of the group. The phylogenetic relationships based on Maximum Likelihood (ML) recovered our sequences together with the one from the Department of Tolima with a support of 99.5/100% (Fig. 1, Table 1) within the species of *R.festae* group. Finally, the *R.festae* group clade is also supported by 100/100.

**Table 1.** Genetic distances between species of *R.festae* group. Intraspecific (on the diagonal) and interspecific (below the diagonal) distances based on *p-distance* method for the mitochondrial *16S* rRNA gene.

Species		Accessions	1	2	3	4	5	6	7	8	9	10	11	12	13	14	15	16	17	18	19	20	21
1	***Rhinellakumanday* sp. nov. MHN-Uca-Am 1164**	[**OR680126**]	/																				
2	***Rhinellakumanday* sp. nov. MHN-Uca-Am 1492**	[**OR680127**]	0.000	/																			
3	*Rhinella* sp. 4	[KT221613]	0.000	0.000	/																		
4	Rhinellacf.nicefori	[MW003543]	0.036	0.037	0.053	/																	
5	*Rhinellaruizi*	[MW003567; MW003568]	0.038	0.039	0.054	0.001	0.000																
6	*Rhinellaparaguas*	[MW003552; MW003553]	0.055–0.057	0.056–0.058	0.057–0.063	0.050–0.055	0.051–0.057	0.035															
7	*Rhinellaacrolopha*	[MW003440; MW003441]	0.069–0.070	0.070–0.072	0.079–0.081	0.074–0.075	0.075–0.076	0.083–0.088	0.011														
8	*Rhinellamacrorhina*	[MW003517; MW003518; MW003519; MW003520]	0.080–0.083	0.082–0.085	0.083–0.092	0.076–0.082	0.077–0.083	0.074–0.095	0.074–0.079	0.000–0.022													
9	*Rhinellatenrec*	[MW003593; MW003594]	0.099	0.101	0.095–0.099	0.090–0.092	0.092–0.093	0.090–0.099	0.076–0.088	0.064–0.066	0.000												
10	*Rhinellalindae*	[MW003514; MW003515; MW003516]	0.065	0.066	0.066–0.073	0.060–0.061	0.062–0.063	0.060–0.075	0.060–0.066	0.060–0.067	0.070–0.075	0.000–0.002											
11	*Rhinellaarborescandens*	[MW003449; MW003450; MW003451]	0.063–0.066	0.064–0.067	0.073–0.075	0.066	0.067	0.068–0.077	0.066–0.071	0.063–0.068	0.079–0.081	0.053–0.060	0.000–0.018										
12	*Rhinellarostrata*	[AF375533]	0.066	0.069	0.080	0.080	0.082	0.096–0.099	0.083–0.084	0.075–0.094	0.094–0.098	0.074–0.083	0.079–0.080	/									
13	*Rhinellafestae*	[KR012624; DQ158423]	0.066–0.067	0.068–0.069	0.074–0.078	0.064–0.070	0.065–0.071	0.074–0.080	0.075–0.083	0.054–0.076	0.077–0.087	0.052–0.067	0.060–0.070	0.017–0.019	0.000								
14	*Rhinellachullachaki*	[MW493261]	0.081	0.081	0.081	0.079	0.081	0.085–0.094	0.083–0.087	0.102–0.104	0.126	0.086	0.069–0.075	0.070	0.075–0.078	/							
15	*Rhinellalilyrodriguezae*	[MW003509; MW003510; MW003511]	0.082–0.085	0.084–0.088	0.080–0.083	0.078–0.082	0.079–0.083	0.084–0.091	0.088–0.094	0.080–0.094	0.097–0.109	0.073–0.083	0.061–0.064	0.089–0.096	0.077–0.083	0.058	0.001–0.023						
16	*Rhinellachavin*	[DQ158441]	0.079	0.081	0.091	0.084	0.085	0.086–0.090	0.081–0.082	0.087–0.099	0.091–0.094	0.075–0.082	0.074–0.078	0.090	0.076–0.085	0.070	0.068–0.072	/					
17	Rhinellacfmultiverrucosa	[MW003540]	0.072	0.074	0.086	0.080	0.082	0.083–0.089	0.080–0.081	0.084–0.095	0.086–0.091	0.072–0.079	0.071–0.077	0.090	0.074–0.083	0.065	0.067–0.071	0.000	/				
18	*Rhinellayanachaga*	[MW003603; MW003604]	0.064–0.066	0.066–0.068	0.085–0.087	0.078–0.080	0.079–0.081	0.077–0.090	0.079–0.085	0.086–0.097	0.094–0.096	0.064–0.081	0.068–0.071	0.092–0.094	0.078–0.083	0.059–0.061	0.067	0.043–0.046	0.040–0.042	0.002			
19	*Rhinellamanu*	[MW003522; MW003523]	0.087–0.089	0.089–0.091	0.084–0.085	0.072–0.073	0.073–0.075	0.073–0.092	0.089–0.090	0.090–0.097	0.097–0.102	0.081–0.084	0.071–0.072	0.074	0.072–0.074	0.085	0.088–0.091	0.084	0.082	0.079–0.082	0.000		
20	*Rhinellanesiotes*	[MW003541; MW003542]	0.079–0.082	0.081–0.084	0.083–0.084	0.080–0.082	0.081–0.083	0.077–0.095	0.090–0.095	0.077–0.080	0.094–0.099	0.080–0.084	0.072–0.078	0.074–0.082	0.068–0.077	0.089–0.091	0.079–0.084	0.082–0.088	0.082–0.085	0.085–0.089	0.048–0.050	0.006	
21	*Rhinellatacana*	[MW003589; MW003590; MW003592]	0.076–0,084	0,078–0,084	0,085–0,088	0,080–0,083	0,082–0,085	0,081–0,106	0,090–0,094	0,079–0,096	0,097–0,102	0,088–0,092	0,072–0,081	0,072–0,085	0,074–0,079	0,087–0,089	0,085–0,091	0,087–0,093	0,083–0,090	0,079–0,090	0,050–0,058	0,022–0,025	0,001–0,009

Based on our analyses, we concluded that the specimens from the Central Cordillera of Colombia in the departments of Caldas and Tolima do not represent any of the described *Rhinella* species. Therefore, we assert that they belong to a new, undescribed species that we thoroughly document and describe in this study as *Rhinellakumanday* sp. nov.

### ﻿Systematics

**Family Bufonidae Gray, 1825**

**Genus *Rhinella* Fitzinger, 1826**

#### ﻿ Rhinella kumanday sp. nov.

Taxon classificationAnimaliaAnuraBufonidae

E4B19944-A13B-54FD-8CF8-162DADF9B3AF

https://zoobank.org/3392CAEB-0277-419E-ADAE-F03B1B1D697A

**Figure 2.**
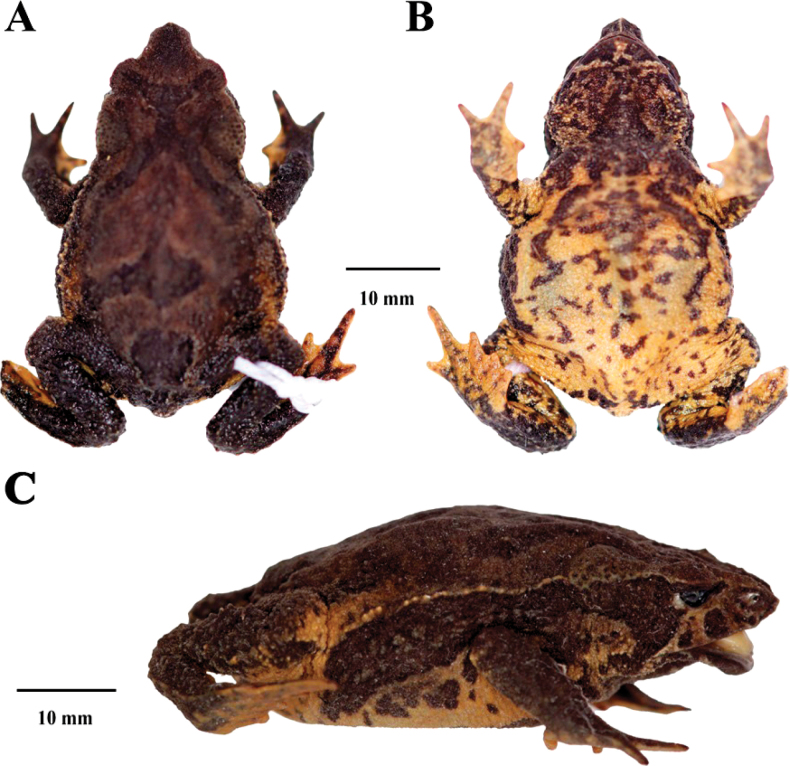
Holotype of *Rhinellakumanday* sp. nov. (MHN-UCa-Am 1164, adult female), SVL 39.4 mm, in preservative **A** dorsal view **B** ventral view **C** lateral view.

**Figure 3.**
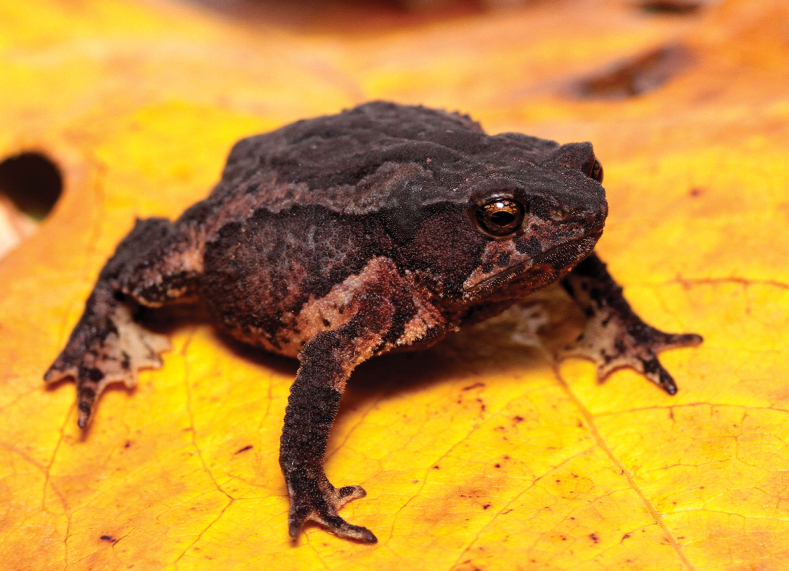
Female (MHN-UCa-Am 1698; paratype) of *Rhinellakumanday* sp. nov. in life (SVL 35.01 mm).

**Figure 4.**
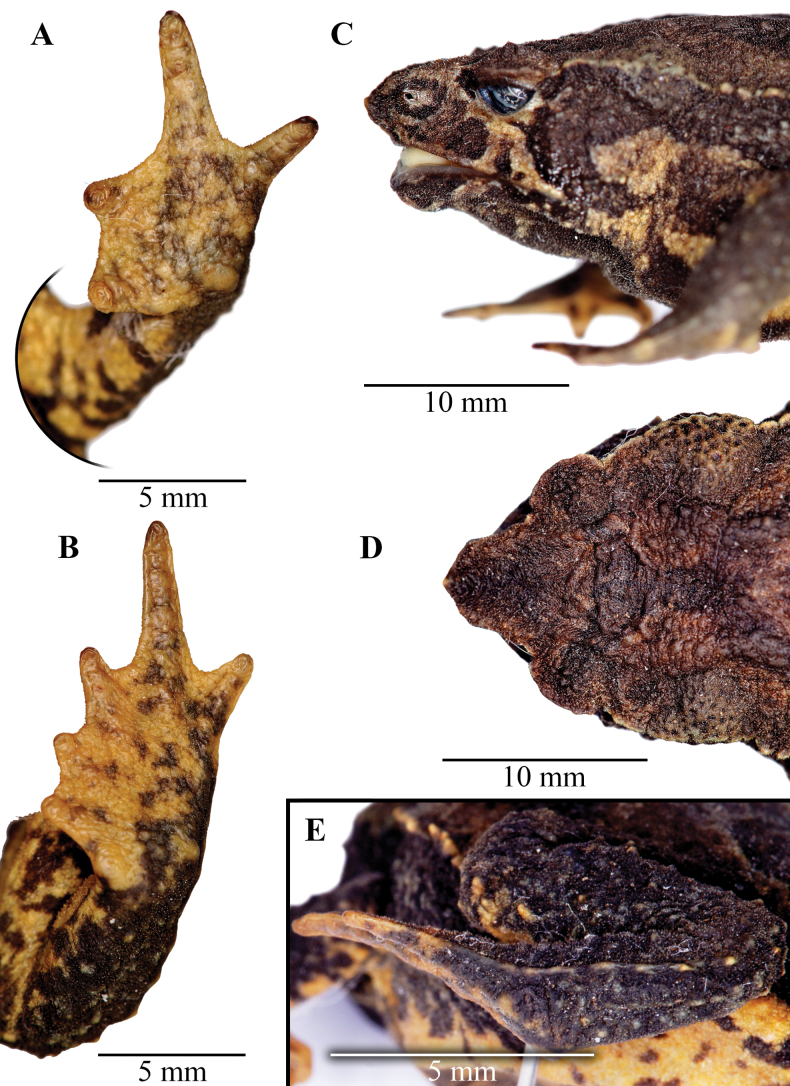
Head, hand, and foot of the holotype of *Rhinellakumanday* sp. nov. (MHN-UCa-Am 1164) **A** dorsal view of head **B** lateral view of head **C** ventral view of right hand **D** ventral view of right foot **E** tarsal fold on the left foot.

**Figure 5.**
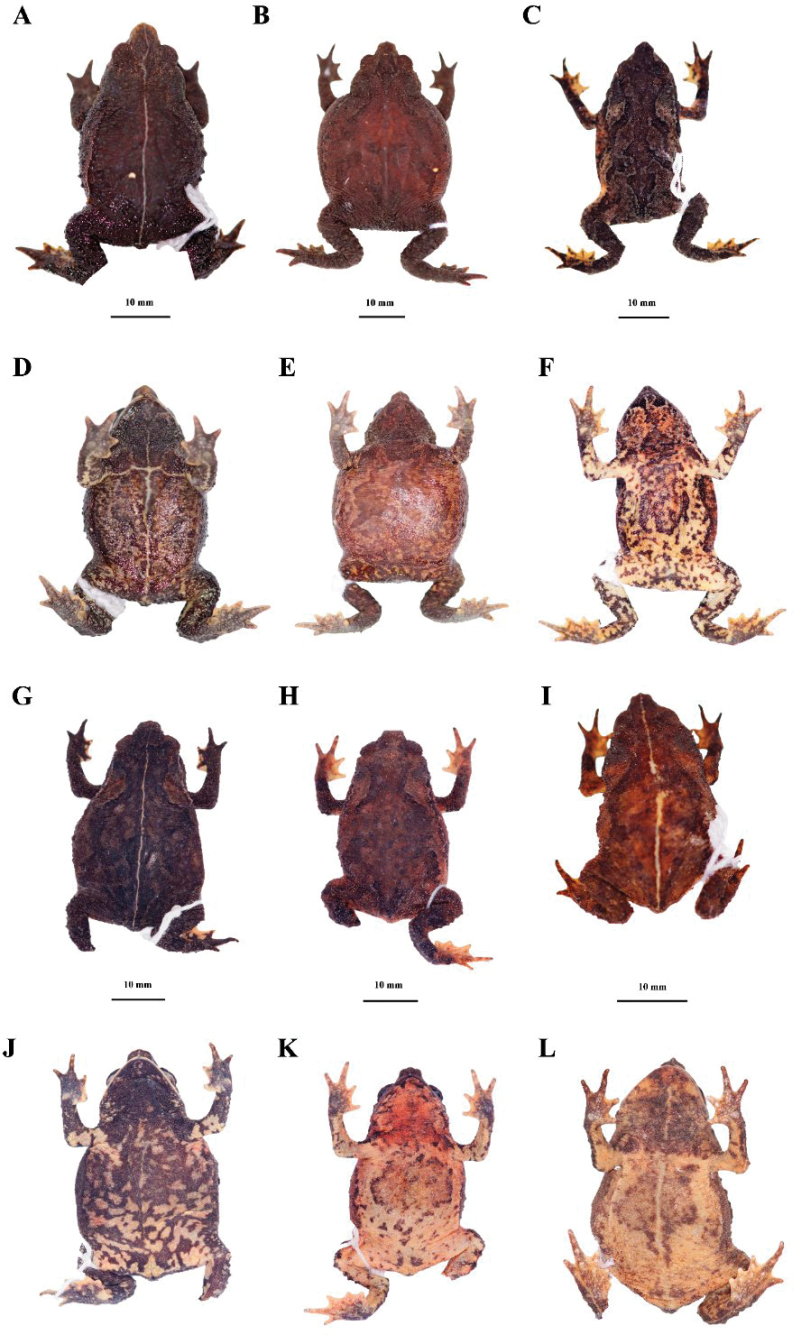
Paratypes and referred specimens of *Rhinellakumanday* sp. nov. in preservative **A, B, C, G–I** dorsal views (MHN-UCa-Am 199, 198, 1698, 1718, 1717, 421, respectively) and ventral views **D–F, J–L** (MHN-UCa-Am 199, 198, 1698, 1718, 1717, 421, respectively).

**Figure 6.**
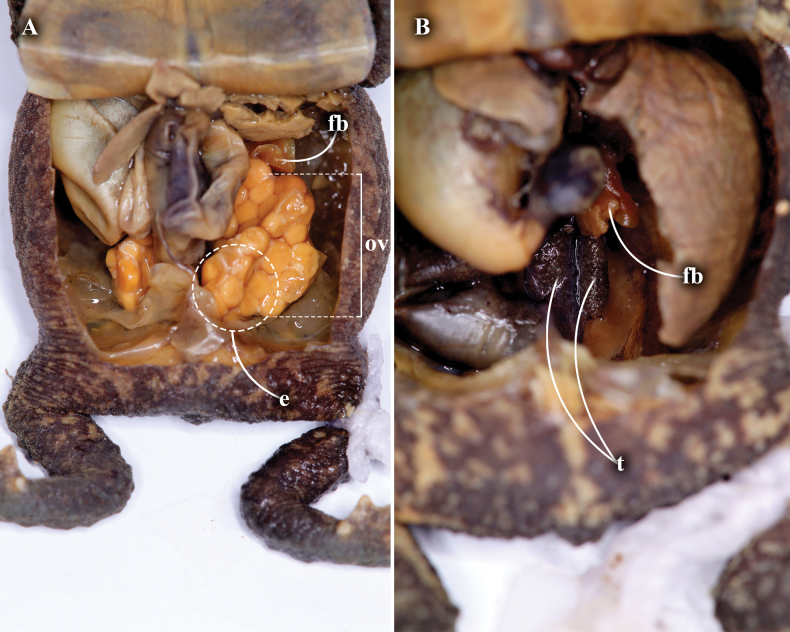
Internal sexual anatomy of *Rhinellakumanday* sp. nov. **A** female (MHN-UCa-Am 198) **B** male (MHN-UCa-Am 199). Abbreviations: **fb** fat bodies; **ov** ovary; **e** ovarian eggs; **t** testes.

Figs 2 , 3 , 4 , 5 , 6 Suggested English name: Kumanday Beaked Toad Suggested Spanish name: Sapo picudo del Kumanday

Rhinella sp.: Rojas-Morales et al. 2014: 87, 89.
Rhinella sp. C.: Machado et al. 2016: 687, 688, 689, 690, 692, table 1.
Rhinella sp. C: Cusi et al. 2017: 26, 42, 43.
Rhinella sp. Gómez-Salazar et al. 2017: 78, table 1.
Rhinella sp. C (= Rhinellasp.acrolopha group sensu Grant and Bolívar-G. 2014): Cusi et al. 2017: 27, table 1.
Rhinella sp.: Rojas-Morales and Marín-Martínez 2019: 13266, 13269, 13270, 13271, 13274, image 4B, fig. 4, appendix 1
Rhinella sp. 4: Pereyra et al. 2021: 40, 63, 65, 112, 131, fig. 13, table 10, appendix 1, appendix suppl. data 2, suppl. data 3.5, suppl. data 4.5, map 7.
*R*[*hinella*]. sp. “gr. acrolopha”: Pereyra et al. 2021: 112, 131, appendix 1, appendix 2.
R[*hinella*]. sp. ‘C’: Castillo-Urbina et al. 2021: 182, 184, fig. 1.

##### Type material examined.

***Holotype*.**MHN-UCa-Am 1164 (adult female, Fig. 2), from Torre 4, Reserva Forestal Protectora Bosques de la CHEC, municipality of Manizales, Department of Caldas, Colombia (5.0266, -75.39299), 2730 m collected by Jose J. Henao-Osorio (JJHO) on 24 October 2019. ***Paratypes*** (*n* = 6): 3 females, 2 males, 1 indeterminate sex (Figs 3, 4). Two adult males (MHN-UCa-Am 196, 199) and one adult female (MHN-UCa-Am 198), from Reserva Forestal Protectora Bosques de la CHEC, municipality of Villamaría, Department of Caldas, Colombia (4.99833, -75.39194; 2954 m) collected by Oscar López-Castrillón (OLC-005), Luisa F. Galvis (LFG-009) and Juan M. Pérez (JMP-010) respectively, on 12 October 2012. An adult female (MHN-UCa-Am 1698) from El Cedral, Reserva Forestal Protectora Bosques de la CHEC, municipality of Villamaría, Department of Caldas (5.027671, -75.414618, 2695 m) collected by Héctor F. Arias-Monsalve on 5 March 2023. one female (MHN-UCa-Am 1718) from Reserva Ecológica Río Blanco, municipality of Manizales, Department of Caldas (5.074302, -75.43353, 2705 m,) collected by L. Santiago Caicedo-Martínez (SCM-139) on 25 May 2023. An individual with unknown sex (MHN-UCa-Am 1492, stained skeleton) from the Reserva Forestal Protectora Bosques de la CHEC in Gallinazo, municipality of Villamaría, Department of Caldas (4.99833, -75.39194, 2954 m) collected by Héctor F. Arias-Monsalve (HFA-364) on 23 February 2022.

##### Other specimens examined.

Three females, the first (MHN-UCa-Am 421) from Vereda Montaño municipality of Villamaría, Department of Caldas (4.99213, -75.45565, 2433 m) collected by Gustavo Gonzales-Durán (GGD-055) on 18 May 2009; the second (MHN-UCa-Am 1717) from Reserva Ecológica Río Blanco, municipality of Manizales, Department of Caldas (5.074720, -75.434795, 2667 m) by SCM (138) on 25 May 2023; and the third (MHN-UCa-Am 1802) collected at the Reserva Ecológica Río Blanco.

##### Diagnosis.

A moderate-sized species of the *Rhinellafestae* group sensu Pereyra et al. (2021), and which can be distinguished from other members of the genus by the following combination of characters: males SVL 36.4–37.8 mm (x– = 37.1; *n* = 2); females SVL 32.5–40.1 mm (x– = 37.1; *n* = 5). (1) seven presacral vertebrae; (2) sacral vertebrae no fused with the coccyx, but fused with the urostyle; (3) sagittal ridge present; (4) snout long, protuberant and directed anteroventrally; (5) canthal crest present but weak; (6) preorbital crest present; (7) supraorbital present; (8) postorbital crest weak; (9) pretympanic crests weak; (10) supratympanic crest distinct; (11) parietal crest present; (12) dorsal surface with scattered tubercles, small and round with some conical ones; (13) parotoid glands well developed and ovoid; (14) lateral row of tubercle variable from scattered conical tubercle from the posterior side of the parotoid gland until 2/3 of the lateral space to the groin, to a complete fold of conical tubercles from the posterior part of the parotoid glands to the groin; (15) skin on dorsal surface of limbs with many warts and conical tubercles; (16) finger I smaller than II; (17) fingers and toes moderate webbed, digits long; (18) subarticular tubercles diffuse, barely evident in some individuals (19) many supernumerary tubercles, small, round and low; (20) modal webbing in hand: I 1–2 II 1–2 III–2–2 IV; in foot: I 0–0 II 0–2 III 1–3 IV 3–1 V; (21) males without vocal slits; (22) nuptial pads absent in males; (23) testes small and black; (24) coloration in life: dorsum light brown, in some cases with few dark spots and/or cream middorsal line; flanks dark yellow with many grey and dark mottling; venter creamy yellow with variable size marks dark brown; iris golden with irregular dark brown marks.

##### Description of the Holotype

**(Figs 2, 4).** Adult female (SVL = 39.43 mm; Fig. 2), body robust; head triangular in dorsal view, protruding and sharp in lateral view; head wider than long (HW 1.5 times HL) narrower than body; HW and HL 34.1% and 22.8% of SVL, respectively. Snout acuminate, triangular in the tip; SL 59.9% of the HL; the distance from the nostril to the tip of the snout is equal to the distance from the anterior margin of the eye to the nostril (2.6 mm); snout protruding and sharp; upper jaw directed beyond the lower. Snout protruding with a sagittal ridge between the upper lip and the point of the snout. *Canthusrostralis* elevated forming a weak canthal crest, concave in dorsal view; loreal region concave; nostril round, small and protuberant, no visible from dorsal view; eye diameter more than half of the interorbital distance (ED/IOD = 0.53); ED longer than the distance between eye and nostril (ED/E-N = 1.25). Canthal crest present but weak; preorbital crest non evident but present; supraorbital crest present; postorbital crest weak; supratympanic crest present, distinct, expanded laterally; pretympanic crest present. Tympanic annulus and tympanic membrane absent. Parotoid glands subtriangular, large, almost two times ED (PL/ED = 1.88). Skin of the eyelid with abundant small warts of different shapes and sizes, eyelid edge above the eye forming a thick fold. Forearm slender, 25.4% of SVL; forearm skin bearing abundant subconical warts and smaller conical tubercles along the entire surface. Hands long, slender, hand length 25.1% of SVL; hands densely covered by minute tubercles in the entire dorsal surface; fingers slender and long; relative length of fingers I<II<IV<III; finger tips round; basal webbing between fingers present, webbing formula: I 1–2 II 2–3 III -3–2^+^IV; fingers with lateral fringes; palmar and thenar tubercles distinct, small and ovoid; thenar less than a half of palmar tubercle; palmar surface of the hand with multiple accessory tubercles like warts and minute tubercles barely visible; supernumerary and subarticular tubercles diffuses. Hindlimbs shorts, robust; femur length and tibia length 33.7% and 34.6% of SVL, respectively; entire surface of the hindlimbs covered with abundant warts and conical and subconical tubercles of different sizes foot long, 33.2% of SVL; toes long and slender; relative length of toes I<II<III<V<IV; toes tip round; toes with extensive webbing between toe I and II, moderate in toes III, IV and V, webbing formula: I 0–0 II 0^+^–2- III 1–3- IV 3-–1^+^ V; fingers bearing lateral fringes; tarsal fold present, formed from the postaxial lateral fringe of toe V, in the portion proximal to the heel the tarsal fold is replaced by tubercles; outer metatarsal tubercle barely visible, round, ~ 3× smaller than the round inner metatarsal tubercle; plantar surface with flat warts indistinct, accessory tubercles small, indistinct; supernumerary tubercles indistinct; subarticular tubercles diffuses, round.

Dorsum with scattered subconical, round warts with hard tips and densely covered with minute conical tubercles; lateral row of subconical tubercles from the posterior region of the parotoid gland to insertion of the groin, forming a discontinuous fold. Skin more granular in the flanks with more prominent warts densely distributed. Skin on venter with minute tubercles; chest and gular region more granular than venter. Cloacal opening protuberant, directed posteriorly at mid-level of the thighs. Tongue robust, ~ 2× longer than wide; notched anteriorly, one half free posteriorly. Choana small and ovoid, widely separated. Maxillary, premaxilla and vomerine teeth absent. Measurements of the holotype (mm). SVL: 39.4; HW: 13.4; HL: 9; ED: 3.3; IOD: 6.1; EW: 3.3; EL: 4.3; IND: 4.0; E-N: 3.1; NSD: 2.6; SL: 5.4; FL: 10.0; HNDL: 9.9; FEML: 13.3; TL: 13.6; FOOTL: 13.1; PL: 6.2.

##### Coloration of Holotype in life.

Head mainly brown with some darker zones in the tympanic region; the pupil is circular and the iris appears golden, accompanied by black mottled spots that become more grouped near the sclera. The suborbital area is cream yellowish and extend in two lines of the same color, one ends in the mouth corner, and the second, reach the upper lip. The dorsal surface has a light brown background with a dark brown medial band that start at the interorbital space covering both eyelids and extend medially toward the cloacal sheath, becoming a discontinuous mark in the latter; at the level of the parotoid glands and the middle of the dorsum, the aforementioned band turns into an inverted V-shaped mark. The flanks of the body have a light brown coloration, in which highlights a cream yellowish line that extends from the upper eyelid to the groin that is cream yellowish with few black spots, crossing the parotoid glands and the fine lateral row of tubercles. The ventral surface of the body and posterior and anterior extremities have a cream yellowish background, with the presence of brown mottle marks that are more concentrated at gular region and turn diffuses towards the posterior region of venter. The cloacal opening has a divide coloration, in which the upper region is brown with yellow spots, and the lower region has a cream yellowish with brown marks. The tarsal fold is light yellow and extend from the keel to the distal point of the toe V.

##### Coloration of holotype in preservative

**(Figs 2, 4).** The head has mainly a dark brown coloration; the tympanic region is dark brown; the suborbital region has a yellow mustard color and form two lines of the same color that extends to the mouth corner and the upper lip, respectively. The dorsum has a light brown background with a darker band that start in the interorbital space and covers both eyelids and extends to the cloaca turning in a diffuse mark in this region; at the level of the parotoid glands and the middle of dorsum this band forms an inverted V-shaped mark. The flanks are dark brown almost black with a cream yellowish line that start at the upper eyelid and extends to the groin that has a yellow background with black spotting, crossing the brown parotoid glands. The dorsal surfaces of the extremities are dark brown with cream yellowish marks at the webbing and the tips of the fingers and toes. The ventral surfaces of the body and extremities have brown mottled marks on a yellow mustard background; the brown marks are concentrated in the gular region turning into diffuse marks toward the cloaca. The cloaca has two different colorations, in which the upper region is brown with few yellow spots, and the lower region has a yellow background with brown marks. The tarsal fold is yellow.

##### Variation.

There is variation in the measurements between females and males in which females have a longer size in some structures (Table 2). There is also variation in the degree of the dorsolateral row of prominent tubercles as follow: scattered conical tubercles that does not form a row (MHN-UCa-Am 198–199); lateral row of conical tubercles from the anterior part of the parotid glands to 2/3 of the lateral space to the groin (MHN-UCa-Am 196, 0412); complete lateral row of conical tubercles from the anterior part of the parotid glands to the groin (MHN-UCa-Am 1164). Head triangular in dorsal view pointed at the tip in females (MHN-UCa-Am 198, 421, 1164) or blunt at the tip in males (MHN-UCa-Am 196, 199). The color patterns vary among the type series from light brown with darker markings in the dorsum (MHN-UCa-Am 196), a darker brown dorsum with diffuse dark marks (MHN-UCa-Am 198, Am 421, Am 1717; Fig. 5B, H, I, respectively) to a dark brown, almost black dorsum with indistinct black marks (MHN-UCa-Am 199, Am 1698, Am 1718, Fig. 5A, C and G, respectively; and Am 1164, Fig. 2A). Three individuals have a cream/yellowish longitudinal line in the dorsum (MHN-UCa-Am 199, 421, 1718. Fig. 5A, G, I). The variability in the degree of the dark brown mottling and markings in the venter is high (Fig. 5D–F, J–L); the chest and gular region varies from a light cream yellowish background with diffuse and thin dark brown marks more abundant from chest to the gular region (MHN-UCa-Am 196, 421, 1698, 1717) to a cream colored background with thick and abundant dark brown to black markings along the entire venter and completely dark in chest and gular region (MHN-UCa-Am 198–199, 1718). Two individuals present two light cream lines, one longitudinal from the tip of lower lip to the cloacal opening and the second horizontal from the insertion of the arm (MHN-UCa-Am 199, 421). The females have large eggs with a cream-yellow color and lacking reticulations (Fig. 6A). The males lack nuptial excrescences and vocal slits, the testes are small and present black reticulations (Fig. 6B).

**Table 2.** Measurements taken to the nine individuals of *Rhinellakumanday* sp. nov. housed in the MHN-UCa-Am. For the measurements definitions see Materials and methods. MHN-UCa-Am 421, 1717, and 1802 are not part of the type series.

Measurements	Female holotype MHN-UCa-Am 1164	Female MHN-UCa-Am 198	Juvenile female MHN-UCa-Am 421	Female MHN-UCa-Am 1698	Female MHN-UCa-Am 1717	Female MHN-UCa-Am 1718	Female MHN-UCa-Am 1802	Male MHN-UCa-Am 196	Male MHN-UCa-Am 199
**SVL**	39.4	40.1	32.5	35.01	35.54	37.71	35.98	36.4	37.8
**HW**	13.4	13.7	12.1	12.36	12.51	13.26	11.08	11.6	11.5
**HL**	9	8.7	7.2	9.45	9.87	9.61	8.30	6.1	8.0
**ED**	3.3	3.2	3.5	3.12	3.87	3.59	3.92	3.1	3.2
**IOD**	6.1	5.5	4.4	4.23	4.56	4.84	3.85	5.7	2.8
**EW**	3.3	2.6	3.4	2	3.55	3.21	3.02	2.8	2.7
**EL**	4.3	3.7	4.3	3.19	4.71	4.42	3.35	4.8	4.2
**IND**	4.0	3.8	3.4	3.3	3.64	3.63	3.86	3.5	3.8
**E-N**	3.1	2.4	2.4	2.52	2.82	2.81	–	2.3	2.1
**NSD**	2.6	2.9	2.4	1.63	2.5	2.49	3.3	2.8	2.7
**SL**	5.4	5.2	4.5	4.96	4.93	5.06	–	5.6	5.2
**FL**	10.0	9.0	6.9	8.55	8.36	7.96	10.06	7.1	7.0
**HNDL**	9.9	9.6	7.4	9.39	9.06	8.33	4.59	8.6	8.5
**FEML**	13.3	10.9	9.9	12.6	12.69	12.79	13.32	8.2	9.0
**TL**	13.6	12.8	9.6	11.42	11.44	10.91	14.99	11.3	10.5
**FOOTL**	13.1	11.6	10	12.03	11.67	11.12	14.21	11.1	10.9
**PL**	6.2	5.9	5.7	4.84	4.35	4.7	–	5.8	5.4

##### Osteological description.

The following description is based on two stained and cleared adult female specimens (MHN-UCa-Am 1802, SVL = 35.98 mm; MHN-UCa-Am 1492, SVL = 35.01 mm).

***Cranium*.** Shape of anterior margin of nasal bones are relatively blunt and in contact medially; the anterior margin of the frontoparietal bones are not completely articulated with the posterior margin of nasals making the dorsal surface of sphenethmoid visible; dermal roofing bones heavily ornamented with pits, striations and rugosities; canthal crest blunt; preorbital crest present; supraorbital crest blunt and thick; postorbital crest present but weak; supratympanic crest present, distinct, expanded laterally towards the postorbital crest, but the occipital artery pathway avoids connecting with the supraorbital crest; pretympanic crest weak; parietal crest weak; the occipital artery canal partially cover by bones; otic ramus enlarged in contact with the posterolateral margin of frontoparietal bones; anterior margins of the nasal bones extended beyond the dorsal margins of the alary processes of the premaxillae; alary processes of the premaxillae angled posteriorly to the anterior margin of the premaxillae; the anterior end of the septomaxilla is not developed; the zygomatic ramus of squamosal is free from the ventral ramus; the jaw articulation is opposite to the fenestra ovalis; columella is present but reduced in size and articulated with the palatoquadrate and squamosal; tympanic annulus is absent; frontoparietal extends beyond the lateral margins of the sphenethmoid; the sphenethmoid reaches the palatines; the anterior ramus of the pterygoid articulates along the margin of maxilla and does not contact the palatine; ventral ridge of the palatines absent; medial ramus of the pterygoid is fused with the anterolateral margin of the parasphenoid; the surface of contact is jagged between the medial ramus of pterygoid and parasphenoid alae; anterior margin of the cultriform process of the parasphenoid is broadly rounded anteriorly; bony protrusion at the angle of the jaw absent.

***Vertebral column*.** The axial column consists of seven presacral vertebrae with the neural spine flat or slightly elevated, and vertebrae I–II are fused. The decreasing lengths of the transverse processes and sacrum are: III>IV>Sacrum>V>VII>VI>II; the maximum width of the sacral diapophysis is smaller than the maximum length (maximum length and width of MHN-UCa-Am 1492: 5.36 mm and 3.85 mm, and MHN-UCa-Am 1802: 5.38 mm and 3.74 mm, respectively); the length of the transverse processes of III and VI vertebrae are larger than the length of the transverse process of vertebrae V (maximum length: 8.61 mm, 9.70 mm and 7.18 mm, respectively); transverse process of the VI vertebrae is parallel to the V vertebrae; transverse process of the VII vertebrae is orientated anteriorly in relation to the medial axis of the vertebral column. The anterior edge of the sacral diapophysis is oriented anteriorly to the midline axis of the vertebral column; posterior margin of the sacral diapophyses is relatively smooth; the fusion of the sacrum and the urostyle is distinguished; the urostyle present lateral fringes in dorsal view. Ilium presents a large dorsally directed protuberance and its dorsal crest is present but small; in lateral view, the anteroventral margin of the symphysis of the iliac bone with the iliac axis of the pelvic girdle is perpendicular to the plane of the iliac bone, forming an angle of 90°; ilia shaft lacks the dorsal crest in medial view; in lateral view, the relative contribution of the ischium to the pelvic girdle is not evident, but the contribution of the ilium to the pelvic girdle is observed, indicating a possible fusion between the ischium and the pubis; the postventral crest of acetabular expansion of ischium is well developed.

***Pectoral girdle*.** The pectoral girdle is composed of various bones and cartilaginous elements, which may exhibit different degrees of mineralization; sternum presents mesosternum and xiphisternum of reduced size, occupying a small lower portion of coracoid, where a degree of mineralization can also be observed; free epicoracoid, partially ossified on the closest edge to the coracoid; each protocoracoid continues through the epicoracoid, reaching the upper part of the clavicle, and it expands laterally until it reaches the distal end where the clavicle articulates with the scapula; the omosternum is absent; a moderate-sized foramen is observed in the upper part of the glenoid cavity, probably caused by the medial union of the scapula, clavicle, and coracoid; clavicle small (~ 5 mm in length in MHN-UCa-Am 1802); well-ossified scapula being 2/3× clavicle length; the scapulae are wider at their lateral ends; anterior and posterior margins of each scapula are concave; distal end of each scapula has a bicondylar articulation; the most distal region of the pectoral girdle is formed by the cleithrum and the suprascapula; degree of ossification between the cleithrum and the suprascapula can vary, making it difficult to establish a boundary between the structures; the cleithrum is more ossified towards the anterior margin and extends laterally forming an incomplete rectangle (3/4 of the plate); the posterior border is cartilaginous and lobulated.

***Forelimbs*.** The humerus is the longest bone of the forelimb; the caput humeri (glenoid epiphysis) is rounded; the eminentia capitate is visible as is a large, rounded structure in the distal epiphysis; the shaft has a well–developed ventral ridge that originates near the proximal head of the humerus and extends to 2/3 of the humerus. A poorly developed proximo–medial ridge is observed; the fossa cubitalis ventralis is narrow and inconspicuous; the radius and ulna are completely fused medially into a single structure that shows a longitudinal sulcus (sulcus intermedius) from the distal head to the head to the middle of diaphysis; olecranon (proximal head of the ulna), and capitulum (proximal head of the radius) are conspicuous and form a concave articulation surface with the eminentia capitata. The autopodium has a set of carpal bones (ulnare, radiale, element Y, distal carpal 2, distal carpal 5–4–3, four metacarpals and their corresponding phalanges (II to V) plus two ossified prehallical elements; elongated metacarpals (IV>V>III>II); relative length of fingers is IV>V>III>II; the ultimate phalanx of the manual digits is pointed; the phalangeal formula is 2–2–3–3; in ventral view, manus presents a pad-like ossified structure.

***Hind limbs*.** The femur is ~ 35% of SVL, has a robust appearance and slightly sigmoidal shape, accompanied by a rounded caput femoralis that fits into the acetabulum of the pelvic girdle; in lateral view, a slight femoral crest is observed and occupies 1/4 of the femur length; tibia and fibula are fused, only distinguishable by the presence of the sulcus intermedius; femur (~ 12 mm) is slightly longer than the tibia-fibula (~ 11 mm); the tibia-fibula present equal length and are fused at the distal and proximal epiphyses. The autopodium consist of a series of tarsal elements (tibia-fibula, and two distal elements), five metatarsal elements with their corresponding phalanges (I–V), and two ossified prehallical elements; element Y is located proximally to metatarsal I and articulating medially with the proximal elements of prehallux; two voluminous elements likely represent fused element Y + distal tarsal 1, and distal tarsals 2 and 3, or element Y and distal tarsals 1–3; the last phalanx of the toes of the hind leg is pointed; phalangeal formula is 2–2–3–4–3, and the relative length of the toes is IV>III>V>II>I.

##### Distribution.

**Figure 7.**
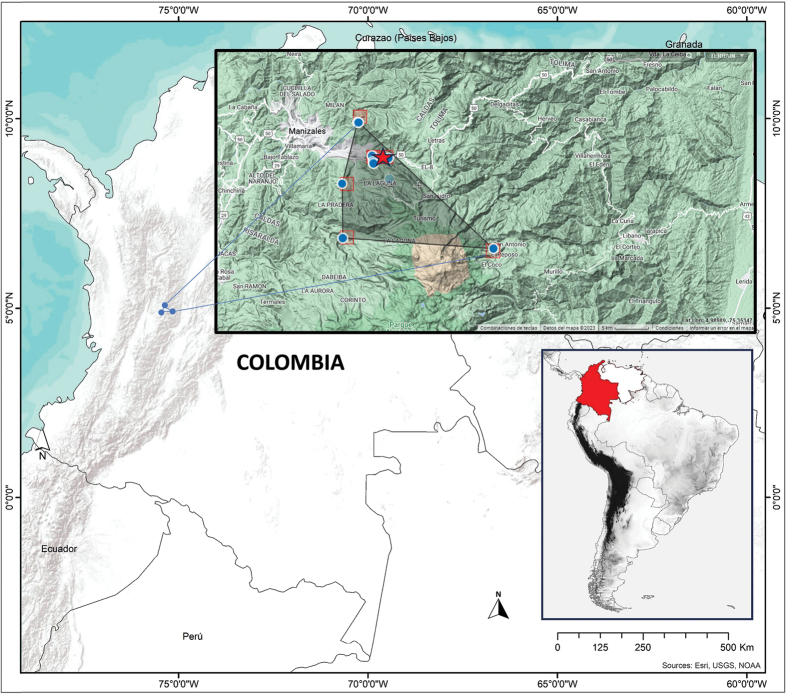
Distribution of *Rhinellakumanday* sp. nov. Records obtained from specimens deposited in the Colección de Anfibios of the Museo de Historia Natural of the Universidad de Caldas (MHN-UCa-Am) and the specimen (TG 2115) mentioned by Machado et al. (2016) and Pereyra et al. (2021). Red star indicates the type locality.

**Figure 8.**
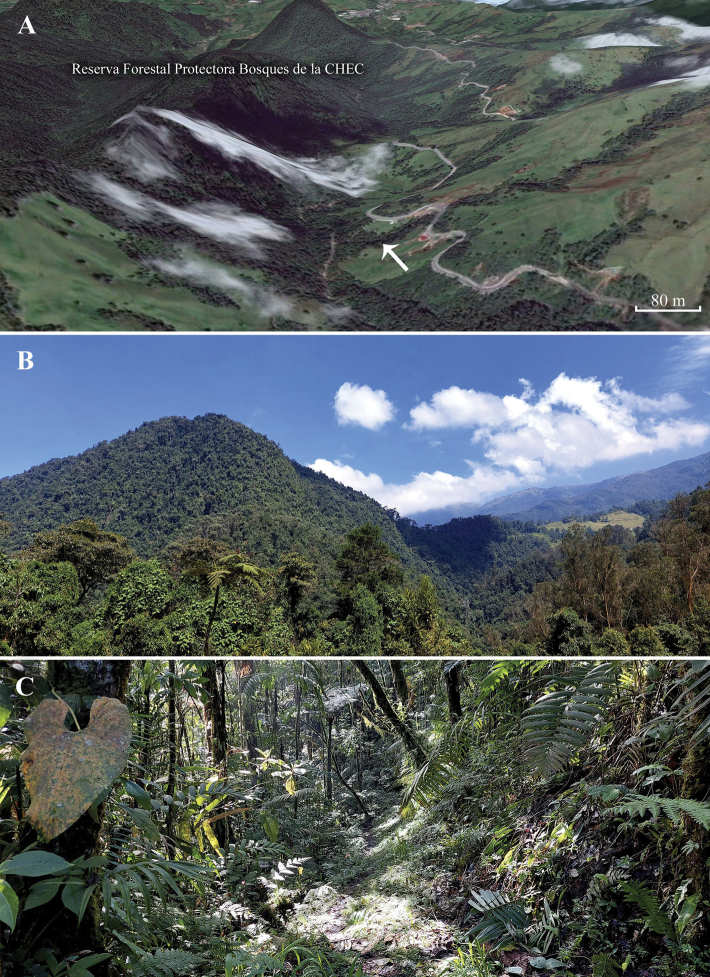
The Andean cloud forests of Manizales and Villamaría, Caldas **A** Google Earth© image with the type locality (arrow) of *Rhinellakumanday* sp. nov. **B** general landscape of the Reserva Forestal Protectora Bosques de la CHEC **C** collection site inside the forest.

*Rhinellakumanday* sp. nov. is distributed in the Central Cordillera of Colombia in an elevational range from 2404 to 3690 m (Fig. 7). It inhabits Andean and high-Andean vegetation (Fig. 8) of the Cauca and Magdalena Montane Forests (sensu Dinerstein et al. 2017). Records of this toad are confined to the area adjacent to Los Nevados Natural Park, which is in the northernmost volcanic belt of the Central Cordillera.

##### Etymology.

The name “kumanday” means “white beautiful”, a word given by the indigenous Quimbaya to the snow-covered volcano that towers over the Central Cordillera in the coffee growing region of Colombia.

##### Comparisons with other species.

According to our genetic results (Fig. 1), the most related species of the *festae* group to *Rhinellakumanday* sp. nov. are *R.paraguas*, *R.ruizi*, and *R.nicefori* with which are grouped. However, *R.kumanday* sp. nov. differs from *R.paraguas* and *R.nicefori* by the presence of a well-defined tarsal fold; moreover, differs from *R.paraguas* by having a smaller body size (Table 3), the presence of a well-defined parietal crest, and the absence of nuptial excrescences in males (parietal crest absent, nuptial excrescences present in *R.paraguas*; Grant and Bolívar-G. 2014). Also, *R.kumanday* sp. nov. differs from *R.ruizi* by the presence of a well-defined sagittal ridge between the point of the snout and the superior lip (absent in *R.ruizi*; Grant 2000), and the number of presacral vertebrae (seven in *R.kumanday* sp. nov. vs eight in *R.ruizi*; Grant and Bolívar-G. 2014). Moreover, the new species differs from other species of the *festae* group such as *R.acrolopha*, *R.chullachaki*, *R.festae*, *R.lindae*, *R.macrorhina*, *R.rostrata*, and *R.truebae* by the aforementioned presence of the tarsal fold (absent in those species); from *R.acrolopha*, *R.festae* and *R.macrorhina* by the presence of low cranial crest (prominent cranial crest in the three species; Trueb 1971); from *R.tenrec*, *R.lindae* and *R.truebae* by having a smaller body size (Table 3; Lynch and Renjifo 1990; Rivero and Castaño 1990). Also, *Rhinellakumanday* sp. nov. differs from *R.tenrec* by the less acute snout (very acute in *R.tenrec*), less presacral vertebrae (seven vs eight in *R.tenrec*), the fusion between sacral and the urostyle (not fused in *R.tenrec*), and the elevational distribution (> 2000 m in *R.kumanday* sp. nov. vs ≤ 1300 m in *R.tenrec*). *Rhinellakumanday* sp. nov. also differs from *R.truebae*, *R.chavin*, *R.lilyrodriguezae*, *R.manu*, *R.multiverrucosa*, *R.nesiotes*, *R.tacana*, and *R.yanachaga* by the absence of the tympanum and annulus tympanic (present in those species).

**Table 3.** Measurements (in mm) of some species of the *R.festae* group distributed in the Colombian Andes. The information was obtained from the literature and include minimum and maximum range, mean and standard deviation for *R.paraguas*. In the case of *R.kumanday*, the male data do not show the variation as there were only two males. For *R.tenrec* the complete dataset of measurements is given only for the female holotype, and for *R.truebae*, the species is only known from one female individual.

Measurements	*R.kumanday* females n = 5	*R.kumanday* males n = 2	*R.paraguas* females	*R.paraguas* males	*R.tenrec* females	*R.lindae* females	*R.lindae* males	*R.truebae* females	*R.macrorhina* females	*R.nicefori* females	*R.nicefori* males	*R.rostrata* males
**SVL**	35–40.1, (37.3, ± 2,1)	37.1	40.6–51.4, (45.1, ±0.7)	31.3–41.7, (35.5, ±0.8)	54.7–60.8, 56.7	62.2	26.9	65.9	51.4	32.9	31.9	42
**HW**	11.1–13.7, (12.7, ± 1.0)	11.6	13.1–16.7, (14.9, ±0.2)	10.2–14.6, (11.8, ±0.3)	22.3	–	–	20.5	18.8	11.4	11.1	14.3
**HL**	8.3–9.9, (9.2, ±0.6)	7.1	12.2–14.4, (13.0, ±0.1)	9.6–11.8, (10.6, ±0.2)	20.1	23.8	9.0	20.8	18.2	10.1	10.0	13.6
**ED**	3.1–3.9, (3.5, ±0.4)	3.2	–	–	5.00	–	–	6.6	4.8	3.2	3.3	4.6
**IOD**	3.9–6.1, (4.9, ± 0.8)	4.3	4.3–5.8, (5, ±0.1)	3.1–5.0, (4.2, ±0.2)	10.7	11.0	4.0	9.9	8.2	4	4	6.5
**EW**	2–3.6, (2.9, ±0.6)	2.7	–	–	3.8	–	–	4.5	–	–	–	–
**EL**	3.2–4.7, (4.0, ±0.6)	4.5	4.8–5.7, (5.3, ±0.1)	3.9–4.7, (4.4, ±0.1)	4.8	4.0	2.0	–	–	–	–	–
**IND**	3.3–4, (3.7, ±0.24)	3.7	4.0–4.8, (4.5, ±0.1)	3.5–4.3, (3.9, ±0.1)	–	–	–	–	–	–	–	–
**E–N**	2.4–2.8, (2.7, ±0.3)	2.2	2.6–3.5, (3.0, ±0.1)	2.0–2.9, (2.5, ±0.1)	6.0	6.1	3.0	6.5	4	2.2	1.9	3.3
**NSD**	1.6–3.3, (2.6, ±0.6)	2.7	2.3–3.5, (3.1, ±0.1)	2.3–3.1, (2.7, ±0.1)	–	–	–	–	4	1.7	1.9	2.4
**SL**	4.9–5.4, (5.1, ± 0.2)	5.4	5.4–6.4, (6.1, ±0.1)	4.7–5.8, (5.2, ±0.1)	–	3	1.5	–	–	–	–	–
**FL**	7.9–10.1, (8.9, ±0.9)	7.0	10.1–13.3, (11.2, ±0.2)	7.4–10.6, (8.6, ±0.2)	–	–	–	–	–	–	–	–
**HNDL**	4.6–9.9, (8.5, ±1.9)	8.5	11.1–13.5, (12.1, ±0.2)	7.3–10.2, (8.8, ±0.2)	–	–	–	–	–	–	–	–
**FEML**	10.9–13.3, (12.6, ±0.9)	8.6	–	–	–	23.25	10.0	–	–	–	–	–
**TL**	10.9–14.9, (12.5, ±1.6)	10.9	13.3–16.9, (14.8, ±0.2)	9.8–12.8, (11.4, ±0.3)	21.3	23.35	10.5	23.8	16	9.0	10.2	15.3
**FOOTL**	11.1–14.21, (12.3, ±1.2)	11	13.3–18.2, (15.8, ±0.3)	9.5–13.5, (11.8, ±0.3)	20.1	27.5	10.5	29.5	26	16.1	15.00	25.1
**PL**	4.4–6.2, (5.2, ± 0.8)	5.6	–	–	–	–	–	11.9	–	–	–	–

##### Conservation status.

*Rhinellakumanday* sp. nov. is only known from 12 localities in the montane forests of both slopes of the Central Cordillera in the departments of Caldas and Tolima, Colombia. The calculation of its extent of occurrence (EOO) using the minimum convex polygon method, as recommended by the International Union for the Conservation of Nature computed with GeoCAT (Bachman et al. 2011), results in an EOO of 208 km^2^. This limited distribution area strongly suggests that the species should be classified as Endangered; however, population dynamics are still unknown. The observed specimens of *R.kumanday* sp. nov. show irregular temporal occurrences and are typically associated with occasional encounters. This new species is considered rare within its distribution area. Recent field surveys conducted in the type locality and surrounding areas have resulted in low capture success rates. Additionally, our knowledge of the species’ natural history, distribution, and reproductive behavior remains incomplete, which raises concerns about its vulnerability. The Parque Nacional Natural Los Nevados and the Reserva Forestal Protectora Bosques de la CHEC, are likely protecting population of this species, but mining activities such as the Tolda Fría project might have adverse effects on the species. Records of *Rhinella* sp. from the departments of Quindío and Risaralda (e.g., Castaño et al. 2017), require further review to confirm whether or not they belong to *R.kumanday* sp. nov.

##### Natural history.

*Rhinellakumanday* sp. nov. presents terrestrial and crepuscular habits. This species has been observed to be associated with leaflitter or under rotten log during early morning hours and twilight (Rojas-Morales and Marín-Martínez 2019 as *Rhinella* sp.). It has been recorded in secondary forests within the Andean ecosystems of the Central Cordillera in the departments of Caldas and Tolima (Fig. 8). The vegetation structure of the locations includes plants from genera such as *Brunellia* Ruiz & Pavón, 1794 (Brunelliaceae), *Chamaedorea* Willd., 1806 (Arecaceae), *Saurauia* Willdenow, 1801 (Actinidiaceae), *Oreopanax* Decaisne & J.E. Planchon, 1854 (Araliaceae), *Cyathea* Sm., 1793 (Cyatheaceae), *Juglans* L., 1753 (Junglandaceae), *Croton* L., 1753 (Euphorbiaceae), and *Ombrophytum* Poepp. ex Endl. (Balanophoraceae). *Rhinellakumanday* sp. nov. has been found at the edges of creeks and streams inside the forest, as well as near streams close to the Pan-American Road in Caldas. Based on observations of seven individuals, Escobar-Lasso and González-Duran (2012, as *Rhinella* sp.) described the defensive behavior of *R.kumanday* sp. nov. The behavior included thanatosis or death feigning. The only known natural predator of this species is the False-coral snake *Erythrolampruslamonae* (Dunn, 1944) (pers. obs.). *Rhinellakumanday* sp. nov. shares its habitat with other species, including the bufonid *Osornophrynepercrassa* Ruiz & Hernández, 1976, in parts of its distribution. It is also likely to be sympatric with *Atelopusquimbaya* Ruiz & Osorno, 1994, which inhabits similar environments in the altitudinal band of the Los Nevados area. However, there have been no records of the latter species since 1997.

Regarding diet, based on dissection of three stomachs of preserved specimens (MHN-UCa-Am 198, 1492, 1802), we found three invertebrate prey items belonging to Coleoptera (Curculionidae and one unidentified) and Acari. The reproductive biology is not documented. We have not seen tadpoles or amplectant pairs; however, three preserved gravid females with an unknown gravity period (MHN-UCa-Am 198, 1802, and GGD-001) contained 96 (diameter 1.82 ± 0.19; *n* = 10), 38 (1.81 mm ± 0.21; *n* = 10), and 81 eggs (1.40 mm ± 1.50; *n* = 10), respectively. The eggs presented mostly a yellowish cream coloration in preservative. This color condition has also been reported in preserved specimens of *R.acrolopha*, *R.chavin*, *R.festae*, *R.macrorhina*, and *R.nicefori* (Trueb 1971; Lehr et al. 2001). The calls of *R.kumanday* sp. nov. are unknown, although we tried to record them in captivity without success.

## ﻿Discussion

We present compelling evidence encompassing morphological, genetic, and osteological traits supporting the recognition of a new species of *Rhinella* in the Central Andes of Colombia. Over the past decade, a series of studies conducted in montane and subparamo forests of the municipalities of Manizales and Villamaría, Department of Caldas, as well as in the municipality of Murillo, Department of Tolima, have consistently alluded to the presence of an undescribed species (Rojas-Morales et al. 2014; Machado et al. 2016; Gómez-Salazar et al. 2017; Rojas-Morales and Marín-Martínez 2019; Pereyra et al. 2021). Our results confirm these previous observations and, in addition, the affiliation of *R.kumanday* sp. nov. with the *R.festae* group. The formal description of *R.kumanday* sp. nov. underscores the remarkable diversity of the genus which now comprises 23 species in Colombia and 93 globally (Acosta-Galvis 2023; Frost 2023). However, the extent of diversity within the genus is still underestimated (Pereyra et al. 2021).

The small number of specimens deposited in biological collections, as well as paucity of collaborative research efforts among scientists interested in this group of toads may have been the cause of the delayed description of this species. Recognizing the vulnerability of many *Rhinella* species within the *festae* group (IUCN 2023), *R.kumanday* sp. nov. is key for the implementation of localized conservation initiatives, as previously undertaken or recommended for other Andean species (Burbano-Yandí et al. 2015). Of the 23 species of *Rhinella* distributed in Colombia, eight are listed as threatened (IUCN 2023), and seven of these threatened species belong to the *R.festae* group: *R.rostrata* (Critically Endangered - EN); *R.acrolopha*, *R.lindae*, *R.nicefori*, and *R.tenrec* (Endangered - EN); and *R.macrorhina* and *R.ruizi* (Vulnerable – VU; IUCN 2023). Beyond Colombian borders, the species of this group face similar threats with four species listed as threatened: *R.nesiotes*: (VU); *R.arborescandens*, *R.chavin* and *R.yanachaga* (EN; IUCN 2023). This means that 50% of the group´s recognized and named species (11 of 21) are considered threatened. Five additional species are included in the Data Deficient category. Increasing field efforts may reveal more threatened species within the genus. In the case of *R.kumanday* sp. nov., we suggest it should be assessed as Endangered due to the extent of its occurrence spanning only 208 km^2^. This species appears to be restricted to the Andean forests of both slopes of the Central Andes in the departments of Caldas and Tolima based on observations of our research team aimed at locating and evaluating the current status of other endangered species such as *Atelopusquimbaya*. During this ongoing project, new individuals of *R.kumanday* sp. nov. have been discovered (MHN-UCa-Am 1717-18).

Based on morphological traits including the snout projected, cranial crest relatively developed, tympanic membrane and annulus tympanic absent (Pereyra et al. 2021:11), *R.kumanday* sp. nov. aligns with the internal clade previously recognized as the *R.acrolopha* group but now part of the *R.festae* group. The lack of tympanic structures is a prevalent trait among species in the *R.festae* group and other *Rhinella* species (Pereyra et al. 2021; Castillo-Urbina et al. 2021). The absence of tympanic structures is linked to a communication mechanism based on the reception of environmental vibrations (Castillo-Urbina et al. 2021). Consequently, exploring the acoustics behavior of the earless *R.kumanday* sp. nov., and data on reproductive aspects such as mating and the morphological traits of the tadpoles are essential for a deeper understanding of the natural history of this species. However, the high yolk concentration as suggested by the mostly yellowish cream coloration of the eggs could indicate that this species has direct development as observed in other amphibian species (de Lima et al. 2016). Reproductive ecology and behavior are important aspects to study in the near future for these poorly known toads. Lehr et al. (2021) stated that *R.manu* is often found far away from the water, suggesting they also may reproduce through direct development. Although *R.kumanday* sp. nov. inhabits near of creeks and streams within the Andean forest, it might also direct-development.

Finally, the Andes play a pivotal role in both the diversification and conservation of amphibians, with new species being discovered every year (Sepúlveda-Seguro et al. 2022). Nonetheless, the Andean and sub-Andean forests in Colombia are highly threatened with only ~ 25% of the original area remaining (Armenteras and Villareal 2003; Etter et al. 2017). Consequently, evaluating the effects of deforestation and the expansion of the agricultural-livestock frontier on small and restricted species such as those within the *R.festae* species group, including the new species described here, is imperative. Furthermore, phylogeographic studies including the Colombian species of this group are relevant for understanding the underlying dynamics influencing their current distribution.

## Supplementary Material

XML Treatment for Rhinella kumanday

## ﻿Additional information

### Author ORCIDs

Luis Santiago Caicedo-Martínez https://orcid.org/0000-0002-9564-5703

Jose J. Henao-Osorio https://orcid.org/0000-0002-8618-8539

Héctor Fabio Arias-Monsalve https://orcid.org/0000-0003-0783-2611

Julián Andrés Rojas-Morales https://orcid.org/0000-0002-3312-8022

Paula A. Ossa-López https://orcid.org/0000-0002-9079-4988

Fredy A. Rivera-Páez https://orcid.org/0000-0001-8048-5818

Héctor E. Ramírez-Chaves https://orcid.org/0000-0002-2454-9482

### Data availability

All of the data that support the findings of this study are available in the main text or Supplementary Information.

## Supplementary materials

Supplementary material 1
Estimates of divergence between sequences based on *p-distance* method for the mitochondrial *16S* rRNA gene
Data type xlsx Explanation note Accessions in bold correspond to this study. https://binary.pensoft.net/file/1009444Copyright notice: This dataset is made available under the Open Database License (http://opendatacommons.org/licenses/odbl/1.0/). The Open Database License (ODbL) is a license agreement intended to allow users to freely share, modify, and use this Dataset while maintaining this same freedom for others, provided that the original source and author(s) are credited.Authors: Luis Santiago Caicedo-Martínez, Jose J. Henao-Osorio, Héctor Fabio Arias-Monsalve, Julián Andrés Rojas-Morales, Paula A. Ossa-López, Fredy A. Rivera-Páez, Héctor E. Ramírez-Chaves

Supplementary material 2 Phylogenetic tree of the partial sequences of the 16S gene of the species of *Rhinella* Data type png https://binary.pensoft.net/file/1009445This dataset is made available under the Open Database License (http://opendatacommons.org/licenses/odbl/1.0/). The Open Database License (ODbL) is a license agreement intended to allow users to freely share, modify, and use this Dataset while maintaining this same freedom for others, provided that the original source and author(s) are credited.Luis Santiago Caicedo-Martínez, Jose J. Henao-Osorio, Héctor Fabio Arias-Monsalve, Julián Andrés Rojas-Morales, Paula A. Ossa-López, Fredy A. Rivera-Páez, Héctor E. Ramírez-Chaves

## References

[B1] Acosta-Galvis AR (2023) Lista de los Anfibios de Colombia: An online reference. V.13.2023. Electronic Database. http://www.batrachia.com [Accessed 10 May 2023]

[B2] Armenteras D Villareal F (2003) Andean forest fragmentation and the representativeness of protected natural areas in the eastern Andes, Colombia. Biological Conservation 113(2): 245–256. 10.1016/S0006-3207(02)00359-2

[B3] Bachman S Moat J Hill A de la Torre J Scott B (2011) Supporting Red List threat assessments with GeoCAT: geospatial conservation assessment tool. ZooKeys 150: 117–126. 10.3897/zookeys.150.2109 PMC3234434 22207809

[B4] Beaupre SJ Jacobson ER Lillywhite HB Zamudio K (2004) Guidelines for use of live amphibians and reptiles in field and laboratory research. American Society of Ichthyologists and Herpetologists, Lawrence, 43 pp.

[B5] Blanco MVF Witmer L (2020) A clearing-and-staining procedure for the study of the chondrocranium and other aspects of skeletal development in crocodilian embryos. Vertebrate Zoology 70: 447–454.

[B6] Burbano-Yandí CE Bolívar-G W Velásquez-Trujillo DA González-Colorado AM Ospina-Herrera O (2015) Plan de conservación de la especie endémica de Colombia Sapito de Páramo *Osornophrynepercrassa*, en el departamento de Caldas. Corporación Autónoma Regional de Caldas (Corpocaldas), Manizales, 40 pp.

[B7] Castaño JH Carranza-Quinceno JA Londoño E Roa Cubillos MM (2017) Biodiversidad del Distrito de Conservación de Suelos Campoalegre. v2.3. Corporación Autónoma Regional de Risaralda - CARDER. Dataset/Checklist. 10.15472/0jxeo4

[B8] Castillo-Urbina E Claw F Aguilar-Puntriano C Vences M Köhler J (2021) Genetic and morphologic evidence reveal another new toad of the *Rhinellafestae* species group (Anura: Bufonidae) from the Cordillera Azul in Central Perú. Salamandra (Frankfurt) 57: 181–195. 10.5281/zenodo.4767016

[B9] Chaparro JC Pramuk JB Gluesenkamp AG (2007) A new species of arboreal *Rhinella* (Anura: Bufonidae) from cloud forest of southeastern Perú. Herpetologica 63(2): 203–212. 10.1655/0018-0831(2007)63[203:ANSOAR]2.0.CO;2

[B10] Cochran DM Goin CJ (1970) Frogs of Colombia. Bulletin of the United States National Museum 288: 1–655. 10.5962/bhl.part.6346

[B11] Cusi JC Moravec J Lehr E Gvoždík V (2017) A new species of semiarboreal toad of the *Rhinellafestae* group (Anura, Bufonidae) from the Cordillera Azul National Park, Peru. ZooKeys 673: 21–47. 10.3897/zookeys.673.13050 PMC5523195 28769671

[B12] de Lima AV Reis AH Amado NG Cassiano-Lima D Borges-Nojosa DM Oriá RB Abreu JG (2016) Developmental aspects of the direct-developing frog *Adelophrynemaranguapensis*. Genesis (New York, N.Y.) 54(5): 257–271. 10.1002/dvg.22935 26953634

[B13] Deforel F Duport-Bru AS Rosset SD Baldo D Candioti FV (2021) Osteological Atlas of *Melanophryniscus* (Anura, Bufonidae): A Synthesis after 150 Years of Skeletal Studies in the Genus. Herpetological Monograph 35(1): 1–27. 10.1655/HERPMONOGRAPHS-D-20-00002

[B14] Dinerstein E Olson D Joshi A Vynne C Burgess ND Wikramanayake E Hahn N Palminteri S Hedao P Noss R Hansen M Locke H Ellis EC Jones B Barber CV Hayes R Kormos C Martin V Crist E Sechrest W Price L Baillie JEM Weeden D Suckling K Davis C Sizer N Moore R Thau D Birch T Potapov P Turubanova S Tyukavina A de Souza N Pintea L Brito JC Llewellyn OA Miller AG Patzelt A Ghazanfar SA Timberlake J Klöser H Shennan-Farpón Y Kindt R Lillesø JPB van Breugel P Graudal L Voge M Al-Shammari KF Saleem M (2017) An ecoregion-based approach to protecting half the terrestrial realm. Bioscience 67(6): 534–545. 10.1093/biosci/bix014 28608869 PMC5451287

[B15] Duellman WE Schulte R (1992) Description of a new species of *Bufo* from northern Peru with comments on phenetic groups of South American toads (Anura: Bufonidae). Copeia 1992(1): 162–172. 10.2307/1446548

[B16] Duellman WE Toft CA (1979) Anurans from the Serranía de Sira, Amazonian Perú: Taxonomy and biogeography. Herpetologica 35: 60–70. https://www.jstor.org/stable/3891754

[B17] Dunn ER (1944) A revision of the Colombian snakes of the genera *Leimadophis*, *Lygophis*, *Liophis*, *Rhadinaea*, and *Pliocercus*, with a note on Colombian *Coniophanes*. Caldasia 2: 479–495. https://www.jstor.org/stable/23640977

[B18] Escobar-Lasso S González-Duran GA (2012) Strategies employed by three neotropical frogs (Amphibia: Anura) to avoid predation. Herpetology Notes 5: 79–84.

[B19] Etter A Andrade A Saavedra K Cortés J (2017) Actualización de la Lista Roja de los Ecosistemas Terrestres de Colombia: conocimiento del riesgo de ecosistemas como herramienta para la gestión: An online reference. Electronic Database. http://reporte.humboldt.org.co/biodiversidad/2017/cap2/204/#seccion1 [Accessed 13 May 2023]

[B20] Fouquet A Gaucher P Blanc M Veléz-Rodríguez CM (2007) Description of two new species of (Anura: Bufonidae) from the lowlands of the Guiana Shield. Zootaxa 1663: 17–32.

[B21] Frost DR (2023) Amphibian species of the world: An online reference. Version 6.1. An online reference. Electronic Database. 10.5531/db.vz.0001 [Accessed 13 May 2023]

[B22] Gómez-Salazar JC Ramírez-Castaño VA Guevara G (2017) Vertebrados terrestres de la Reserva Natural de la Central Hidroeléctrica de Caldas –CHEC– (Villamaría, Colombia): Estado del conocimiento. Boletin Cientifico Museo de Historia Natural Universidad de Caldas 21(1): 71–89. 10.17151/bccm.2017.21.1.6

[B23] Grant T (2000) Una nueva especie de *Rhamphophryne* (Anura: Bufonidae) de la Cordillera Central de Colombia. Revista de la Academia Colombiana de Ciencias Exactas, Físicas y Naturales 23: 287–292. https://www.accefyn.com/revista/Vol_23/supl/287-292.pdf

[B24] Grant T Bolívar-G. W (2014) A new species of semiarboreal toad with a salamander-like ear (Anura: Bufonidae: *Rhinella*). Herpetologica 70(2): 198–210. 10.1655/HERPETOLOGICA-D-13-00082R1

[B25] Graybeal A (1997) Phylogenetic relationships of bufonid frogs and tests of alternate macroevolutionary hypotheses characterizing their radiation. Zoological Journal of the Linnean Society 119(3): 297–338. 10.1111/j.1096-3642.1997.tb00139.x

[B26] Guindon S Dufayard JF Lefort V Anisimova M Hordijk W Gascuel O (2010) New algorithms and methods to estimate maximum-likelihood phylogenies: Assessing the performance of PhyML 3.0. Systematic Biology 59(3): 307–321. 10.1093/sysbio/syq010 20525638

[B27] IUCN (2023) The IUCN Red List of Threatened Species. Version 2022-2. An online reference. Electronic Database. https://www.iucnredlist.org [Accessed 14 May 2023]

[B28] Kalyaanamoorthy S Minh BQ Wong TKF von Haeseler A Jermiin LS (2017) ModelFinder: Fast model selection for accurate phylogenetic estimates. Nature Methods 14(6): 587–589. 10.1038/nmeth.4285 28481363 PMC5453245

[B29] Katoh K Standley DM (2013) MAFFT multiple sequence alignment software version 7: Improvements in performance and usability. Molecular Biology and Evolution 30(4): 772–780. 10.1093/molbev/mst010 23329690 PMC3603318

[B30] Lehr E Köhler G Aguilar C Ponce E (2001) New species of *Bufo* (Anura: Bufonidae) from central Peru. Copeia 2001(1): 216–223. 10.1643/­0045-8511(2001)001[0216:NSOBAB]2.0.CO;2

[B31] Lehr E Pramuk JB Lundberg M (2005) A new species of *Bufo* (Anura: Bufonidae) from Andean Peru. Herpetologica 61(3): 308–318. 10.1655/04-90.1

[B32] Lehr E Pramuk JB Hedges SB Córdova JH (2007) A new species of arboreal *Rhinella* (Anura: Bufonidae) from Yanachaga-Chemillén National Park in central Peru. Zootaxa 1662: 1–14.

[B33] Lehr E Cusi JC Rodríguez LO Venegas PJ García-Ayachi LA Catenazzi A (2021) A new species of toad (Anura: Bufonidae: *Rhinella*) from northern Perú. Taxonomy 1(3): 210–225. 10.3390/taxonomy1030015

[B34] Lynch JD Renjifo JM (1990) Two new toads (Bufonidae: *Ramphophryne*) from the Northern Andes of Colombia. Journal of Herpetology 24(4): 364–371. 10.2307/1565051

[B35] Machado JD Lyra ML Grant T (2016) Mitogenome assembly from genomic multiplex libraries: Comparison of strategies and novel mitogenomes for five species of frogs. Molecular Ecology Resources 16(3): 686–693. 10.1111/1755-0998.12492 26607054

[B36] Maciel NM Schwartz CA Colli GR Castro MS Fontes W Schwartz ENF (2006) A phylogenetic analysis of species in the *Bufocrucifer* group (Anura: Bufonidae), based on indolealkylamines and proteins from skin secretions. Biochemical Systematics and Ecology 34(6): 457–466. 10.1016/j.bse.2006.01.005

[B37] Minh BQ Nguyen MA von Haeseler A (2013) Ultrafast approximation for phylogenetic bootstrap. Molecular Biology and Evolution 30(5): 1188–1195. 10.1093/molbev/mst024 23418397 PMC3670741

[B38] Moravec J Lehr E Cusi JC Córdova JH Gvodzkík V (2014) A new species of the *Rhinellamargaritifera* species group (Anura, Bufonidae) from the montane forest of the Selva Central, Perú. ZooKeys 371: 35–56. 10.3897/zookeys.371.6580 PMC3909798 24493953

[B39] Myers CW Duellman WE (1982) A new species of *Hyla* from Cerro Colorado, and other tree frog records and geographical notes from western Panama. American Museum Novitates 2752: 1–32.

[B40] Narvaes P Rodrigues MT (2009) Taxonomic revision of *Rhinellagranulosa* species group (Amphibia, Anura, Bufonidae) with a description of a new species. Arquivos de Zoologia 40(1): 1–73. 10.11606/issn.2176-7793.v40i1p1-73

[B41] Nguyen LT Schmidt HA von Haeseler A Minh BQ (2015) IQ-TREE: A fast and effective stochastic algorithm for estimating maximum-likelihood phylogenies. Molecular Biology and Evolution 32(1): 268–274. 10.1093/molbev/msu300 25371430 PMC4271533

[B42] Noble GK (1920) Two new batrachians from Colombia. Bulletin of the American Museum of Natural History 42: 441–446. http://digitallibrary.amnh.org/handle/2246/1917

[B43] Padial JM Reichle S McDiarmid RW De la Riva I (2006) A new species of arboreal toad (Anura: Bufonidae: *Chaunus*) from Madidi National Park, Bolivia. Zootaxa 1278(1): 57–58. 10.11646/zootaxa.1278.1.3

[B44] Palumbi SR Martin A McMillan WO Stice L Grabowski G (1991) . The simple fool’s guide to PCR, Version 2.0: privately published document compiled by S. Palumbi, 45 pp.

[B45] Peracca MG (1904) Viaggio del Dr. Enrico Festa nell’ Ecuador e regioni vicine. Rettili ed anfibi. Bollettino dei Musei di Zoologia e Anatomia Comparata della R. Universita di Torino 19: 1–41. 10.5962/bhl.part.11596

[B46] Pereyra MO Baldo D Blotto BL Iglesias PP Thomé MTC Haddad CFB Barrios-Amorós C Ibañez R Faivovich J (2016) Phylogenetic relationships of toads of the *Rhinellagranulosa*. (Anura: Bufonidae): a molecular perspective with comments on hybridization and introgression. Cladistics 32(1): 1–18. 10.1111/cla.12110 34732018

[B47] Pereyra MO Blotto BL Baldo D Chaparro JC Ron SR Elias-Costa AJ Iglesias PP Venegas PJ Thomé MTC Ospina-Sarria JJ Maciel NM Rada M Kolenc F Borteiro C Rivera-Correa M Rojas-Runjaic FJM Moravec J De La Riva I Wheeler WC Castroviejo-Fisher S Grant T Haddad CFB Faivovich J (2021) Evolution in the genus *Rhinella*: A total evidence of phylogenetic analysis of neotropical True Toads (Anura: Bufonidae). Bulletin of the American Museum of Natural History 447: 1–155. 10.1206/0003-0090.447.1.1

[B48] Pramuk JB (2006) Phylogeny of South American *Bufo* (Anura: Bufonidae) inferred from combined evidence. Zoological Journal of the Linnean Society 146(3): 407–452. 10.1111/j.1096-3642.2006.00212.x

[B49] Pyron RA Wiens JJ (2011) A large-scale phylogeny of Amphibia including over 2800 species and a revised classification of extant frogs, salamanders and caecilians. Molecular Phylogenetics and Evolution 61(2): 543–583. 10.1016/j.ympev.2011.06.012 21723399

[B50] Rambaut A (2007) FigTree v1.4.3 (Version 1.4.3). A Graphical Viewer of Phylogenetic Trees. http://tree.bio.ed.ac.uk/software/figtree

[B51] Rivera D Prates I Firneno Jr TJ Rorigues MT Caldwell JP Fujita MK (2021) Phylogenomics, introgression, and demographic history of South American true toads (*Rhinella*). Molecular Ecology 31(3): 978–992. 10.1111/mec.16280 34784086

[B52] Rivero JA Castaño CJ (1990) A new and peculiar species of *Rhamphophryne* (Amphibia: Bufonidae) from Antioquia, Colombia. Journal of Herpetology 24(1): 1–5. 10.2307/1564282

[B53] Rojas-Morales JA Marín-Martínez M (2019) Diversity, structure and natural history of amphibian in upper Claro River basin, a buffer zone of the National Natural Park Los Nevados, Central Cordillera of Colombia. Journal of Threatened Taxa 11(3): 13261–13277. 10.11609/jott.4075.11.3.13261-13277

[B54] Rojas-Morales JA Arias-Monsalve HF González-Durán GA (2014) Anfibios y reptiles de la región centro-sur del departamento de Caldas. Biota Colombiana 15: 73–93. https://www.redalyc.org/pdf/491/49140738005.pdf

[B55] Savage JM Heyer WR (1967) Variation and distribution of the tree-frog genus *Phyllomedusa* in Costa Rica, Central America. Beiträge zur Neotropischen Fauna 5(2): 111–131. 10.1080/01650526709360400

[B56] Savage JM Heyer WR (1997) Digital webbing formulae for Anurans: A refinement. Herpetological Review 28(3): 131.

[B57] Sepúlveda-Seguro AA Marín CM Amézquita A García YA Daza JM (2022) Phylogeographic structure suggests environmental gradient speciation in a montane frog from the northern Andes of Colombia. Organisms, Diversity & Evolution 22(3): 803–820. 10.1007/s13127-022-00549-9

[B58] Talavera G Castresana J (2007) Improvement of phylogenies after removing divergent and ambiguously aligned blocks from protein sequence alignments. Systematic Biology 56(4): 564–577. 10.1080/10635150701472164 17654362

[B59] Taylor WR Van Dyke GC (1985) Revised procedures for staining and clearing small fishes and other vertebrates for bone and cartilage study. Cybium 9: 107–109.

[B60] Trueb L (1971) Phylogenetic relationships of certain neotropical toads with the description of a new genus (Anura: Bufonidae). Contribution in Science. Contributions in Science 216: 1–40. 10.5962/p.241202

[B61] Vélez-R CM Ruiz-C PM (2002) A new species of *Bufo* (Anura: Bufonidae) from Colombia. Herpetologica 58(4): 453–462. 10.1655/0018-0831(2002)058[0453:ANSOBA]2.0.CO;2

[B62] Zhang D Gao F Jakovlić I Zou H Zhang J Li WX Wang GT (2020) PhyloSuite: An integrated and scalable desktop platform for streamlined molecular sequence data management and evolutionary phylogenetics studies. Molecular Ecology Resources 20(1): 348–355. 10.1111/1755-0998.13096 31599058

