# Supplementary figures and images for: ﻿A new species of terrestrial toad of the Rhinellafestae group (Anura, Bufonidae) from the highlands of the Central Cordillera of the Andes of Colombia
Source: Zookeys. 2024 Mar 25;1196:149–75. doi: 10.3897/zookeys.1196.114861 (PMC10985400; doi:10.3897/zookeys.1196.114861)

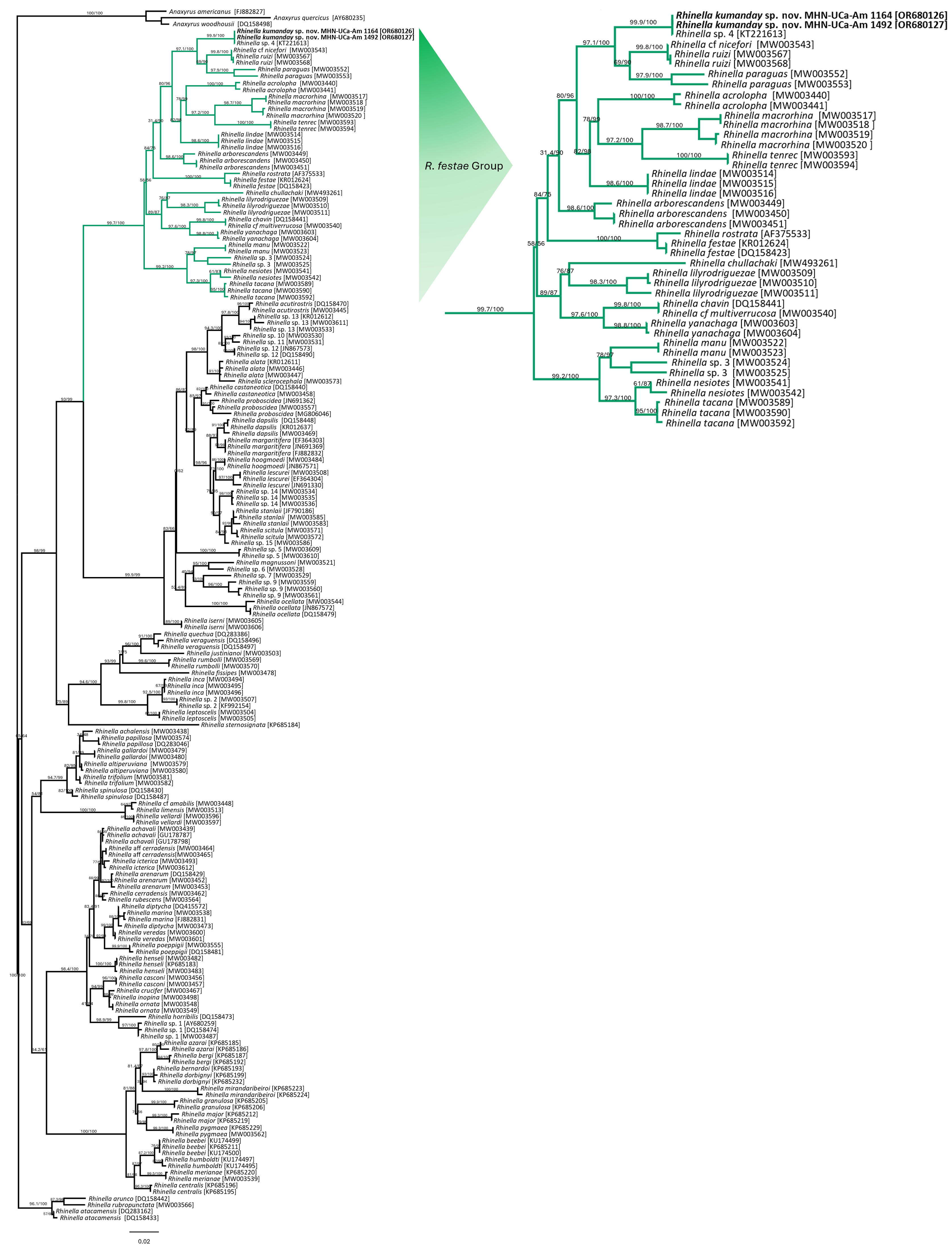

Supplement: Supplementary material 2 — Phylogenetic tree of the partial sequences of the 16S gene of the species of Rhinella [file zookeys-1196-149_article-114861__-s002.png]
